# Environmental-Friendly and Facile Synthesis of Co_3_O_4_ Nanowires and Their Promising Application with Graphene in Lithium-Ion Batteries

**DOI:** 10.1186/s11671-017-2382-4

**Published:** 2017-12-06

**Authors:** Zhiqiang Xu, Wei Liu, Yuanyi Yang, Lijuan Sun, Yi Deng, Li Liao

**Affiliations:** 1 School of Chemical Engineering, Sichuan University, Chengdu, 610065 China; 2 Institute of New Energy and Low-Carbon Technology, Sichuan University, Chengdu, 610065 China; 3 Department of Materials Engineering, Sichuan College of Architectural Technology, Deyang, 618000 China

**Keywords:** Co_3_O_4_ nanowires, Magnetic field, Lithium-ion batteries, Graphene

## Abstract

In this work, we developed an eco-friendly strategy for preparing Co_3_O_4_ nanowires. The process consisted of two steps: controllable synthesis of metal cobalt nanowires followed by a facile air-oxidization step. The 1D nanowire structure with a high aspect ratio was easily achieved via a magnetic-field-assisted self-assembly of cobalt ion complexes during reduction. After air-calcinations, the Co_3_O_4_ nanowires were prepared in large scale and ready to be used as the anode material for lithium-ion batteries. The Co_3_O_4_ nanowires, which possessed a length ranging from 3 to 8 μm with the aspect ratio more than 15, exhibited a reversible lithium storage capacity up to ~ 790 mAh/g when using a small amount of defect-free graphene flakes as conductive additives. The superior electrochemical performances were ascribable to the synergistic “flat-on” effect between the 1D nanowires and the 2D graphene. Therefore, the Co_3_O_4_ nanowire/graphene composite holds promising application for lithium-ion batteries.

## Background

With the fast growing demands for clean and sustainable energy strategy, the electrical energy storage devices are in urgent need for many applications such as electric vehicles and portable electronic devices. Lithium-ion batteries (LIBs) can deliver a relatively high energy density and provide multiple advantages such as long life span, low cost, and good reversibility. Transition-metal oxides have been considered as promising anodes for LIBs due to their abundance, low cost, and high theoretical capacity [1, 2], among which cobalt oxide (Co_3_O_4_) has attracted much attention because of its high theoretical capacity (890 mAh/g) [2–4]. However, the intrinsically low conductivity, large volume and change during cycling, as well as low utilization coefficient of Co_3_O_4_, lead to poor electrochemical performances, hampering its practical application [5, 6].

In recent years, the development of nanoscience and nanotechnology are bringing revolutionary opportunities to further improve the performances of LIB, especially the 1D nanostructures (e.g., nanowires, nanobelts, nanofibers). They have attracted numerous attention owing to their extraordinary electrochemical properties including high surface area, short ion/electro transport pathway, and good capability to accommodate the volume expansion during charge/discharge [5, 7–10].

Although these 1D nanomaterials, such as Co_3_O_4_ nanowires (Co_3_O_4_NWs), are attractive as electrode materials, the synthesis of such nanostructures have raised widespread interest but still remained quite challenging. Many methods for nanowire preparation, including hydrothermal and solvothermal methods [11–13], template-based electro deposition [14], and wet chemical reduction [15], are well developed during past decades. These approaches, however, often involve harsh synthesis conditions such as high pressure/temperature, expensive template, or strong acid (like HF, widely used for dissolving the template), thus hindering the practical application of such nanomaterials. For instance, Dong et al. prepared Co_3_O_4_NWs by heating a pure cobalt foil in the atmosphere, but the reacting time, temperature, and humidity needed to be controlled carefully. The preparation process was complicated and time-consuming [16]. Ji et al. used template-based synthesis method to obtain Co_3_O_4_NWs in the narrow pores of the AAO template, while strong acid and template were indispensable [14]. From Xu et al.’s results, Co_3_O_4_NWs were synthesized through a modified hydrothermal method with high pressure [11]. As found in many literatures, the existing approaches have suffered from many disadvantages, such as complicated operation, difficulty in purification, high cost, and severe environmental pollution. It is of significant importance to develop a new method for synthesizing nanowire structures, with improved scalability, feasibility, and environmental friendliness to overcome the multiple difficulties that has impeded the large-scale application of such nanostructures for long.

Herein, we report a novel, facile, and environmental-friendly method to prepare Co_3_O_4_NWs. A two-step method was adopted in the current study: magnetic-field-assisted synthesis of Co nanowires (CoNWs) and oxidation of CoNWs. With superior efficiency and simplicity, the current method would broaden the electrochemical application of Co_3_O_4_ materials, compared to previous synthesis approaches. To our best knowledge, there is no relevant report regarding this novel synthesis strategy. In this report, the Co_3_O_4_NWs display relatively firm structure with the length-diameter ratio of ~ 15, beneficial for constructing electron/ion transmitting pathways. With the aid of small amount of graphene (3 wt%) acting as conductive support, such Co_3_O_4_NWs/graphene materials have reversible capacities of ~ 790 mAh/g and good rate capabilities when comparing to ordinary Co_3_O_4_ nanoparticles (Co_3_O_4_NPs), making it become a good candidate for lithium-ion battery application.

## Experimental

### Materials

Cobalt (II) chloride hexahydrate (CoCl_2_·6H_2_O, 99 wt%), ethylenediamine tetraacetic acid disodium salt (EDTA-2Na, 99 wt%), sodium hydroxide (NaOH, 98 wt%), sodium chloride (NaCl, 99.5 wt%), potassium chloride (KCl, 99.5 wt%), polyvinylpyrrolidone K30 (PVP (–CH(NCH_2_CH_2_CH_2_CO)CH_2_–)_n_, *M*_w_ = 29,000–35,000), and hydrazine monohydrate (N_2_H_4_·H_2_O, 80 vol%) were purchased from Chengdu KeLong Reagent Co., Ltd. (China), and chloroplatinic acid hexahydrate (H_2_PtCl_6_·6H_2_O, 38 wt%) was purchased from Shenyang Research Institute of Non-ferrous Metals (China). All chemicals were of analytical grade without further purification. All aqueous solutions were prepared with de-ionized water (D.I. water). The commercially available Co_3_O_4_NPs obtained from Shanghai Aladdin Bio-chem Technology Co., Ltd. were used as control group.

### Synthesis of CoNWs

In a typical synthesis, 0.6 mmol CoCl_2_·6H_2_O and 0.6 mmol EDTA-2Na were put into a PTFE beaker of 100 mL capacity, dissolved in 60 mL D.I. water. The pH value of the solution was adjusted to 14 by titration of diluted NaOH, and 0.15 g PVP as surfactant was dissolved in the solution under vigorous stirring. After well dispersed, the beaker was placed between two magnets (in the water bath), with an applied field of 35 mT measured by a HT20 tesla meter, and the water bath was set at 80 °C. When the temperature of the reaction solution reached 80 °C, 0.30 mL N_2_H_4_·H_2_O acted as reductant was added to the above solution for reduction of Co^2+^ and 0.12 mL of 0.0253 mol/L H_2_PtCl_6_·6H_2_O (worked as initiator) were mixed in the solution. The CoNWs formed gradually within 10 min. After reaction, the compound was taken out from the solution with a magnet and washed ultrasonically several times using ethanol and distilled water to remove impurities. Finally, the samples were dried in vacuum freezing drying oven for 12 h.

### Synthesis of Co_3_O_4_NWs

**Fig. 1:**
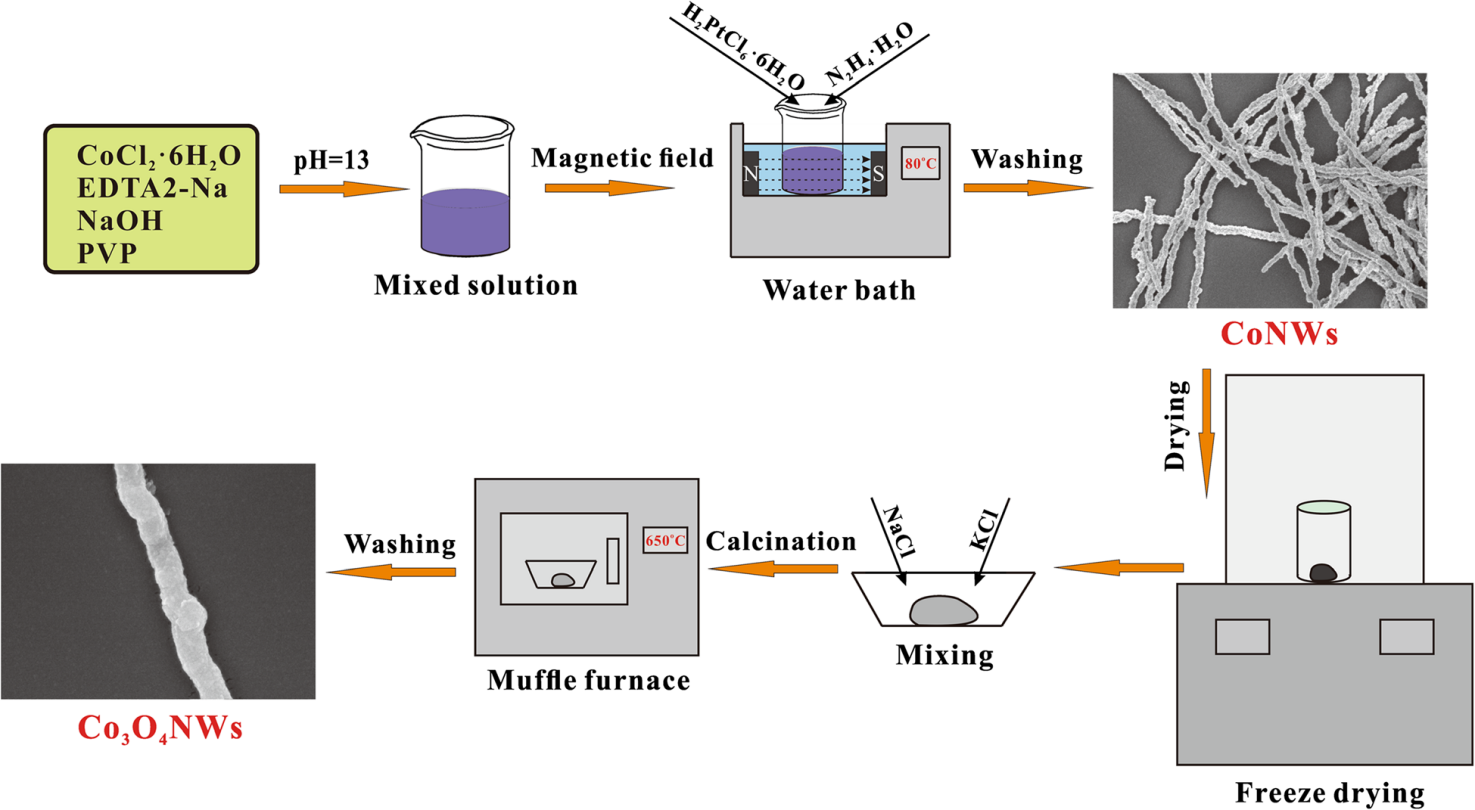
Schematic illustration for preparation Co_3_O_4_NWs

0.01 g of as-prepared sample was put into a porcelain boat mixed with 0.3 g KCl and 0.2 g NaCl uniformly. Then, the mixture was kept in a muffle furnace and heated at 650 °C for 4 h. After the muffle furnace cooled down naturally, the product were taken out and washed by D.I. water three times and dried in the drying oven at 80 °C for 4 h. The formation process of the Co_3_O_4_NWs was shown in Fig. 1.

### Characterization

#### Characterization of Materials

The composition phase of the as-obtained products was verified and compared by X-ray diffraction analysis (XRD, Philips X’Pert Pro MPD) using CuKα as radiation source (λ = 0.154249 nm) at voltage of 40 kV. The diffraction angles (2*θ*) were set between 20° and 90°, with a step size of 4° min^−1^. The phase identification was achieved by comparing the sample diffraction pattern with standard cards in ICDD-JCPDS database.

The morphology of microstructures of samples was characterized by a field emission scanning electron microscope (SEM, JSM-6701F, JEOL, Japan) at an accelerating voltage of 150 kV. All samples were coated with gold before SEM observation.

Transmission electron microscopy (TEM) image and high-resolution TEM (HRTEM) image were taken on a Tecnai-G20 TEM (FEI, USA) for microstructural characterization with an accelerating voltage of 200 kV. Selected area electron diffraction (SAED) was also recorded using the same equipment.

#### Electrochemical Measurements

The electrochemical performances of Co_3_O_4_NWs and Co_3_O_4_NPs were measured based on half coin cells CR2025. The defect-free graphene nanosheets (df-GNS) were produced via a modified liquid-phase exfoliation [17] and then used as conductive component in the electrode. The defect-free graphene with thickness down to ~ 0.5 nm and lateral size of ~ 1 μm was prepared and dispersed in *N*-methyl-pyrrolidone (NMP). The binder-free working electrode was fabricated by coating the mixture slurry, which were composed of active materials (Co_3_O_4_NWs/Co_3_O_4_NPs) and graphene nanosheets (GNS) in a weight ratio of 100:3, onto a copper foil current collector. The active material loading was 0.5~1 mg per cell.

The electrolyte solution was 1 mol/L LiPF_6_ dissolved in a mixture of ethylene carbonate (EC), propylene carbonate (PC), and diethyl carbonate (DEC) with the volume ratio of EC/PC/DEC = 1:1:1. Celgard 2325 membrane was used as the separator. The coin cells were assembled in an argon-filled glovebox where the oxygen and moisture contents were less than 0.1 ppm.

Galvanostatic charge-discharge cycles were tested using a battery testing system (LAND 2001A) within a voltage window of 0.01~3 V. Cyclic voltammograms (CV) of Co_3_O_4_NWs/df-GNS electrode were performed on a commercial electrochemical working station within a voltage range of 0.01~3.0 V at a scan rate of 0.5 mV/s. Electrochemical impedance spectroscopy (EIS) was performed at an open-circuit voltage in a frequency range between 0.1 Hz~100 kHz with a voltage amplitude of 5.0 mV.

## Results and Discussion

**Fig. 2:**
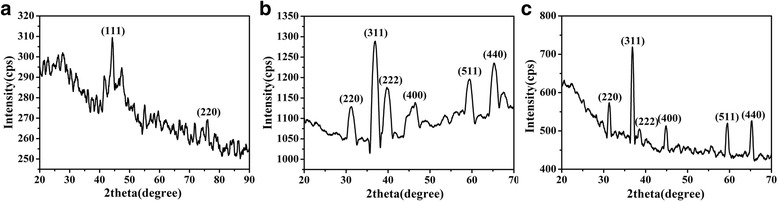
XRD pattern of CoNWs (**a**), Co_3_O_4_NWs (**b**), and Co_3_O_4_NPs (**c**)

The phases of the CoNWs, Co_3_O_4_NWs, and Co_3_O_4_NPs were first investigated by XRD. The Bragg diffraction peaks of them are shown in Fig. 2a–c), respectively. It could be seen that the CoNWs diffraction peaks were well indexed with the reflections of face-centered cubic (fcc) Co (JCPDS No. 15-0806). Typical two characteristic peaks of fcc Co at 2-theta value of 44° and 76° corresponding to Miller indices (111) and (220) were observed, respectively. The Co_3_O_4_NW and Co_3_O_4_NP diffraction peaks were well indexed with the reflections of face-centered cubic (fcc) Co_3_O_4_ (JCPDS No. 15-0806). The recorded diffraction peaks of Co_3_O_4_NPs and Co_3_O_4_NWs at 2-theta = 19°, 31°, 37°, 39°, 45°, 56°, 59° and 65° were well assigned to the (111), (220), (311), (222), (400), (422), (511), and (440) planes of hcp Co_3_O_4_, respectively, with the cell parameter of *a* = 8.084 Å, *b* = 8.084 Å, and *c* = 8.084 Å. Meanwhile, the sharp and high diffraction peaks suggested that the prepared Co_3_O_4_NWs had a high crystalline degree. Next, the average crystalline grain sizes were estimated from the XRD patterns according to the Scherrer formula *D* = *λk*/(*β* cos*θ*) (where *D* is the average crystallite size, *λ* is the X-ray wavelength 0.1542 nm, *k* is the particle shape factor, *β* denotes the angular line width of half-maximum intensity, and *θ* represents the Bragg’s angle) with the values of 18.67 and 25.35 nm for CoNWs and Co_3_O_4_NWs, respectively. It was evident that these values were smaller than the sizes of a single Co particle of nanowires as observed by SEM, which implied that each particle of nanowires was consisted of several crystal grains. No characteristic peaks due to the impurities were detected, indicating that high purity of Co_3_O_4_NWs. It was also surprising to have such high CoNWs-to-Co_3_O_4_NWs conversion yield via simple air-oxidation, which could be assigned to the superior reactivity of CoNWs to oxygen because of its high specific surface area.

**Fig. 3:**
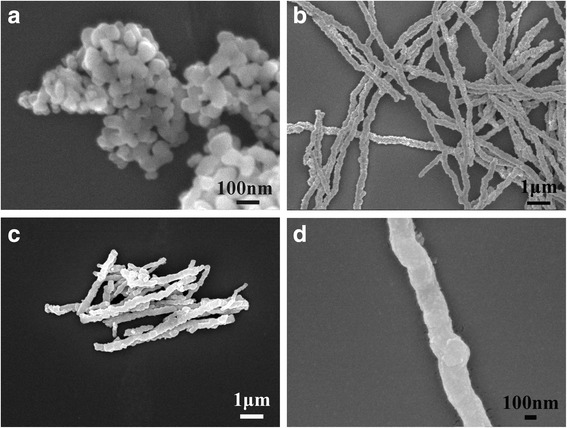
SEM images of Co_3_O_4_NPs (**a**), CoNWs (**b**), and the Co_3_O_4_NWs (**c**, **d**) with different magnifications

The morphologies of the as-obtained samples were characterized by scanning electron microscopy (SEM). The SEM image showed the Co_3_O_4_NPs samples possessed homogeneous diameters of 80 nm and most of the samples were of intact spherical morphology (see in Fig. 3a). Uniform CoNWs with ~ 150 nm in diameter and 20 μm in length were observed as shown in Fig. 3b. A clear necklace-like surface morphology composed of interconnected tiny particles could be seen, verifying the reaction mechanism we proposed above. Moreover, the CoNWs had a robust structural integrity that remained the wire-like shape even after ultrasonication for six times (1 min each time). After rinsing the CoNWs with water and ethanol multiple times, the CoNWs were readily transformed into Co_3_O_4_NWs by simple air-oxidation. The images of Co_3_O_4_NWs (Fig. 3c, d) exhibited the relatively smooth nanowires and remained about 180 nm in diameter, which indicated that the nanowires still maintained wire-like structure after calcinations. So the air-oxidization was a facile and valid approach to obtain Co_3_O_4_NWs.

**Fig. 4:**
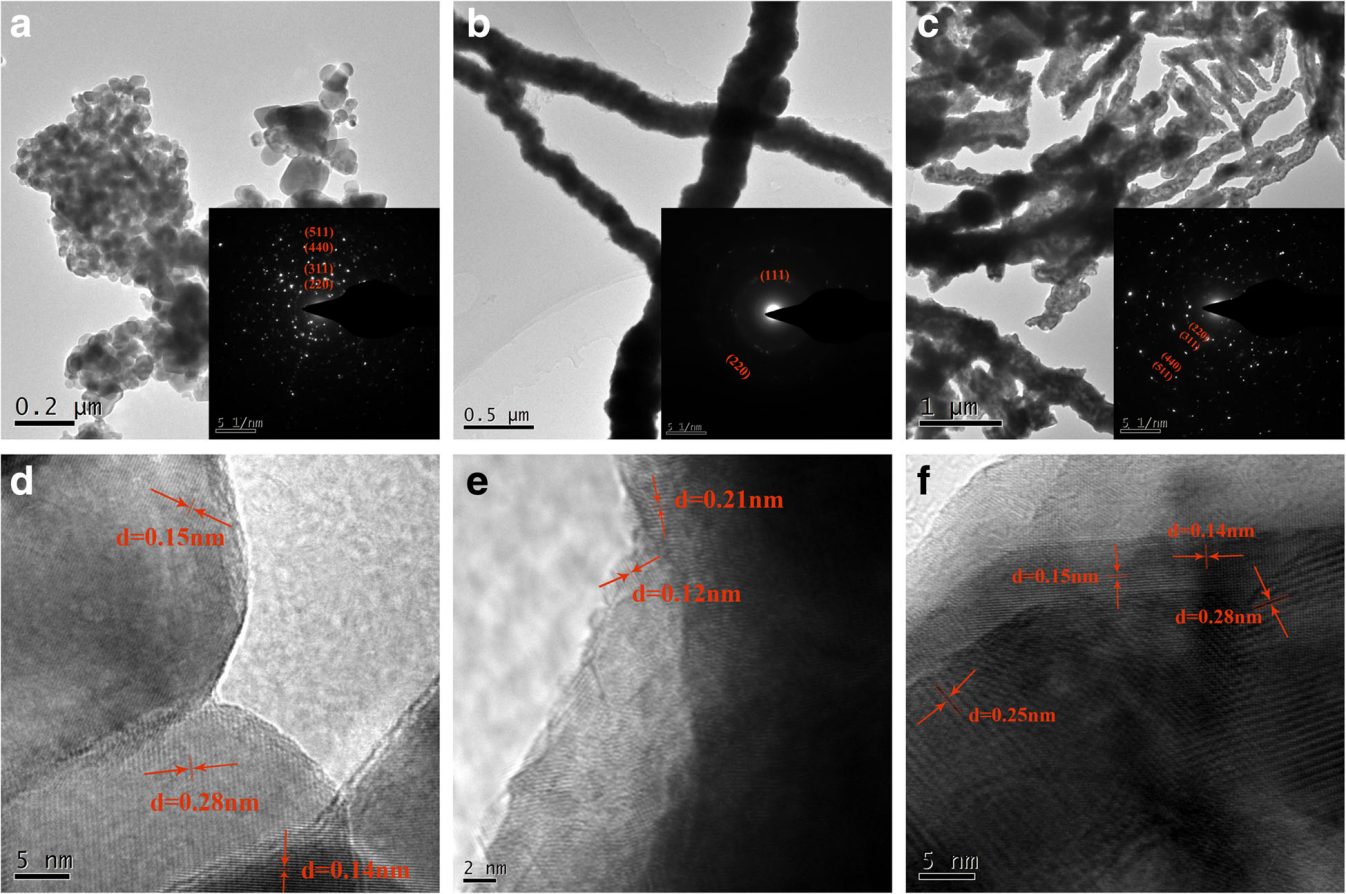
The TEM images of Co_3_O_4_NPs (**a**), CoNWs (**b**), and Co_3_O_4_NWs (**c**). The insets (**a**, **b**, and **c**) showed the selected area electron diffraction (SAED) patterns of the corresponding samples. High-resolution TEM images of Co_3_O_4_NPs, CoNWs, and Co_3_O_4_NWs in (**d**, **e**, and **f**)

The microstructure of Co_3_O_4_NPs, CoNWs, and Co_3_O_4_NWs samples were further investigated by transmission electron microscopy (TEM), selected area diffraction (SAED), and high-resolution transmission electron microscopy (HRTEM), as shown in Fig. 4a–f. Typical TEM images were displayed in Fig. 4a–c, and the morphology and structure of samples were almost consistent with what observed from the SEM images. Furthermore, as the insets in Fig. 4a–c shown, the concentric ring patterns of Co_3_O_4_NPs and Co_3_O_4_NWs (Fig. 4a, c) observed could be assigned to the (200), (311), (440), and (511) planes of Co_3_O_4_, and the concentric ring patterns of CoNWs (Fig. 4b) observed could be attributed to the (111) and (220) planes of Co. The SEAD was exactly identical to the cubic lattice of Co and Co_3_O_4_, respectively, which was consistent with the XRD results. The lattice orientation of the CoNWs in the HRTEM images of Fig. 4e, as the precursor of Co_3_O_4_NWs, had an interplanar distance of 0.12 and 0.21 nm, corresponding to the (111) and (211) planes of Co structure. The lattice spacing (Fig. 4d, f) along the (220), (311), (440), and (511) directions were estimated to be 0.28, 0.25, 0.15, and 0.14 nm, which were close to the standard data of 0.29, 0.24, 0.15, and 0.14 nm.

**Fig. 5:**
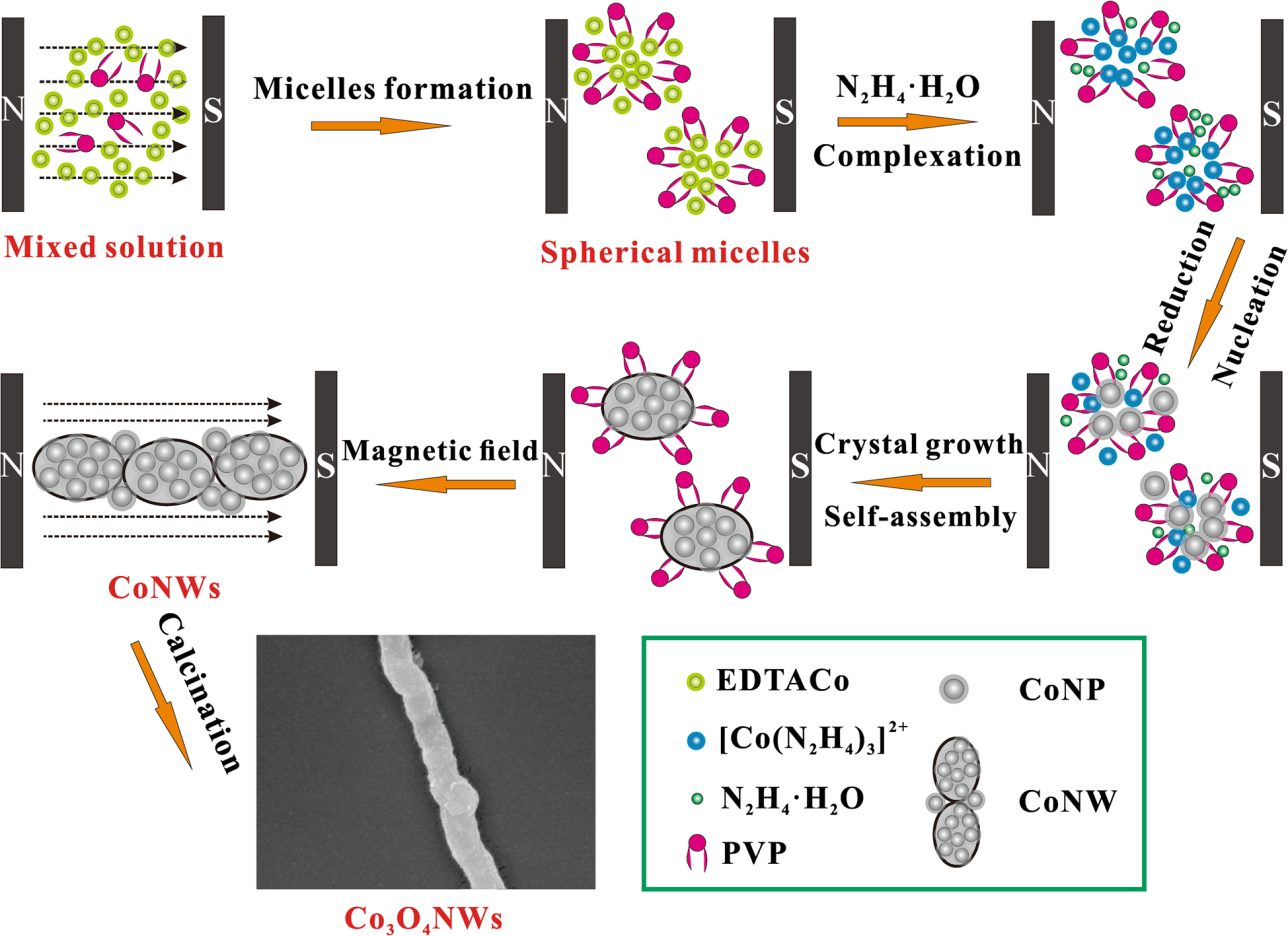
Schematic drawings of the Co_3_O_4_NW formation mechanism assisted by external magnetic field

The CoNWs were prepared via a solution-based reaction route. In Fig. 5, we proposed the possible formation mechanism. In the beginning, EDTA-2Na coordinated with Co^2+^ ions to generate complexes in the solution. Then, the PVP molecules self-assembled to spherical micelles in the water [18, 19], and the Co nuclei were wrapped and possibly absorbed onto the surface of spherical micelles in order to reduce the surface Gibbs free energy. Subsequently, with the introduction of N_2_H_4_·H_2_O into the solution, a portion of N_2_H_4_ ceaselessly replaced the EDTA^2−^ anions and coordinated with Co^2+^ cations to form [Co(N_2_H_4_)_3_]^2+^ complexes, and the rest of them served as reducing agent entered into the mini-reactor and converted [Co(N_2_H_4_)_3_]^2+^ to small Co nanoparticles. Co as well as its compounds was preferred to form micro-spheres according to the previous literatures [20, 21]. Due to the intrinsic magnetic nature of metallic Co, the dipole magnetic moments of the Co atoms are aligned with respect to the external magnetic field direction. As a result, Co nanoparticles will align along the magnetic induction lines to form CoNWs.

**Fig. 6:**
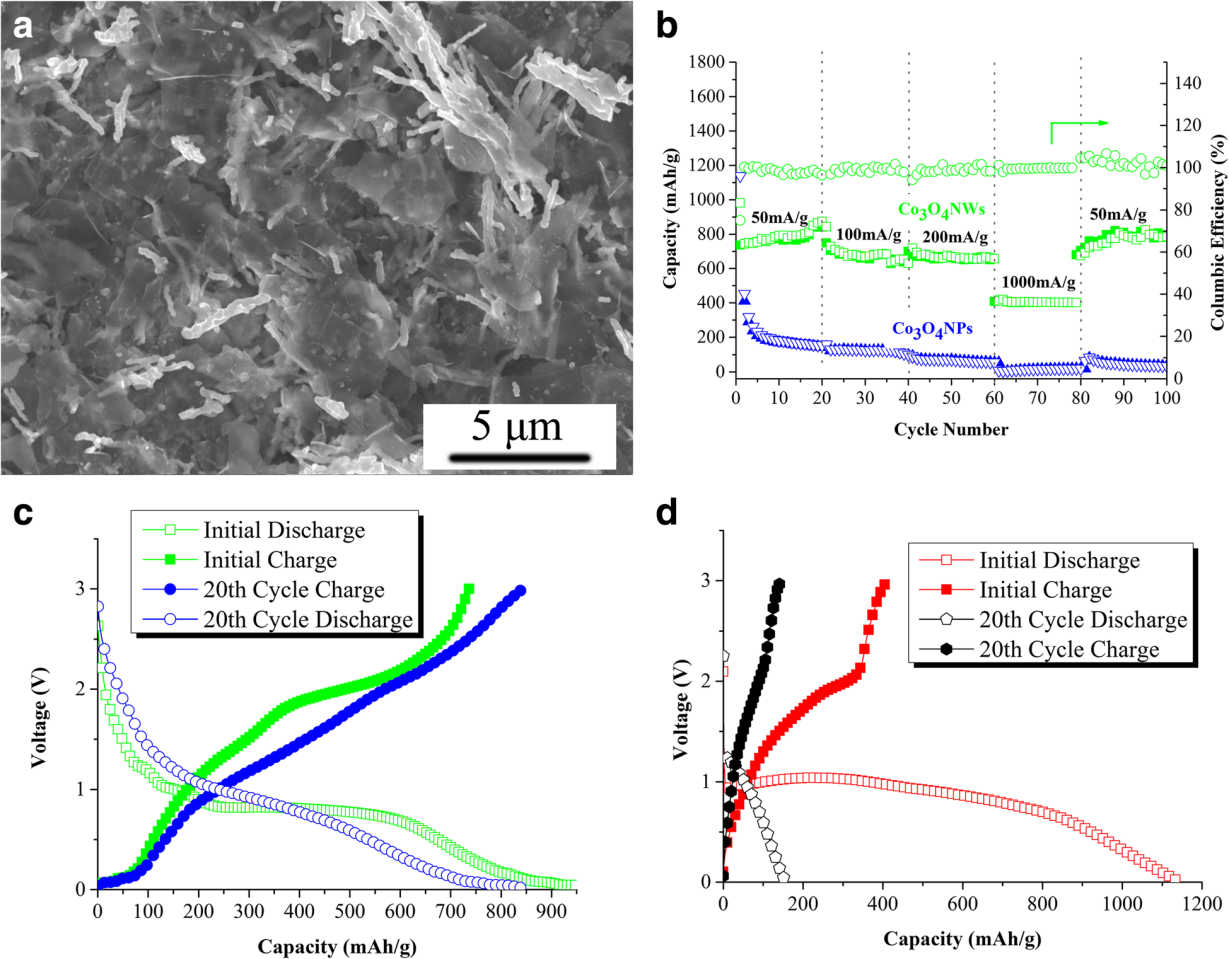
**a** SEM pictures of Co_3_O_4_NWs/df-GNS electrode. **b** Rate capabilities of Co_3_O_4_NWs and Co_3_O_4_NPs under various current densities ranging from 50 to 1000 mA/g. **c**, **d** Typical initial/20th cycle charge and discharge curves of Co_3_O_4_NWs (**c**) and Co_3_O_4_NPs (**d**)

Taking advantage of the 1D geometrical feature of Co_3_O_4_NWs, we prepared the electrodes using a small amount of 2D defect-free graphene (df-graphene) as the conductive support to construct a 1D-2D hybrid structure (The preparation method of df-graphene was described in a previous study [17]). Figure 6a shows the morphology of Co_3_O_4_NWs/df-GNS electrode. For electrode fabrication, the Co_3_O_4_NWs in the powder form were added to the df-GNS/NMP dispersion and then the mixture slurry was sonicated for 10 min before drop-cast on copper foil. As can be seen in Fig. 6b, due to the low-dimensional nature of nanowires and nanosheets, Co_3_O_4_NWs and df-GNS both formed a “flat-on” morphology on the current collector with nanowires embedded in between the nanosheets. This structure can provide multiple advantages: (1) it can prevent these nanomaterials from self-aggregation, especially the self-aggregation and restacking of Co_3_O_4_NWs, which is the main drawback for practical application of such nanostructures; (2) df-GNS can not only offer fast electron pathways but also act as flexible buffer-cushion to accommodate volume change of Co_3_O_4_NWs during charge/discharge; (3) the 1D-2D nanostructure offers numerous pores and nanotunnels to boost ion transport because the external surface area, micropore area, and average pore size of Co_3_O_4_NWs were detected to be 28.554 m^2^/g, 43.697 m^2^/g, 14.682 nm, respectively.

The electrochemical performance of the as-prepared electrodes was evaluated by galvanostatic charge/discharge cycling at various current densities ranging from 50 to 1000 mA/g. As can be seen, the as-prepared Co_3_O_4_NWs/df-GNS electrodes deliver reversible capacities of ~ 790 mAh/g after 20 cycles at current densities of 50 mA/g, little capacity decay was observed during the first 20 charge/discharge cycles (as seen in Fig. 6c). Meanwhile, the Co_3_O_4_NPs/df-GNS electrodes seemed to have relatively high initial capacity of ~ 1130 mAh/g in the first discharge, even higher than that of Co_3_O_4_NWs/df-GNS (~ 980 mAh/g) and the theoretical capacity of Co_3_O_4_ (890 mAh/g). We consider this high initial irreversible capacity is assignable to the formation of solid electrolyte interface (SEI) layer resulting from the decomposition of electrolyte. However, the high initial capacity of Co_3_O_4_NPs/df-GNS seemed to be highly irreversible which decays to ~ 400 mAh/g in the second cycle. After 20 cycles at current densities of 50 mA/g, only ~ 150 mAh/g was observed for Co_3_O_4_NPs/df-GNS electrode (as shown in Fig. 6d). When the current density increased to 100, 200, and 1000 mA/g, the Co_3_O_4_NWs/df-GNS electrode delivered capacities of ~ 680, ~ 650, and ~ 400 mAh/g, respectively, while the Co_3_O_4_NPs/df-GNS electrode exhibits very poor capacity (less than 150 mAh/g at 100–200 mA/g and less than 20 mAh/g at 1000 mA/g).

When the current density comes back to 50 mA/g, a capacity close to 800 mAh/g was obtained in Co_3_O_4_NWs/df-GNS, while the Co_3_O_4_NPs/df-GNS electrode almost lost its ability for lithium-ion storage. The reason for the severe capacity fading of Co_3_O_4_NPs/df-GNS might be attributed to the following factors: (1) large volume change during lithium insertion/extraction, which induced the loss of contact between the active materials and the conductive filler/current collector. During the cycling processes, the Co_3_O_4_NPs electrode gradually lost its electron-transmitting pathway and then eventually resulted in the capacity fading; (2) self-aggregated nanoparticle configuration led to a Li_2_O matrix and/or gel-like polymer layer wrapping around the nanoparticle cluster, which could hinder the ion and/or electron diffusion into the core of the cluster. On the contrary, nanowire/graphene 1D/2D heterostructure in Co_3_O_4_NWs/df-GNS electrode not only constructed the “flat-on” configuration that could accommodate the large volume change but also offered numerous voids and pores to boost ion/electron transmission. As a result, both the cycle and rate performances of Co3O4NWs/df-GNS were significantly improved as compared to Co3O4NPs/df-GNS, maintaining high capacity after 100 cycles.

**Fig. 7:**
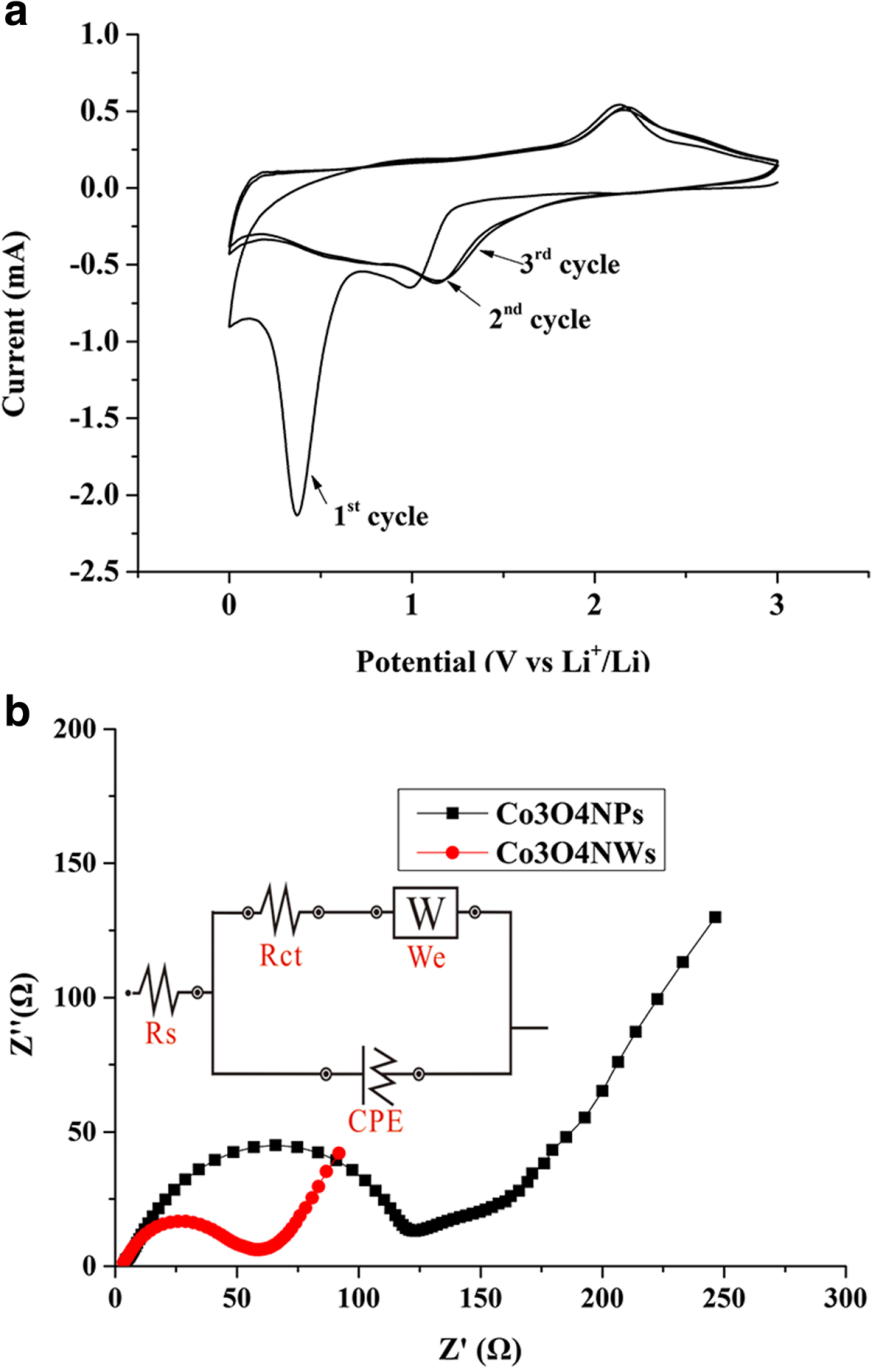
**a** Cyclic voltammograms of Co_3_O_4_NWs/df-GNS electrode at scan rate of 0.5 mV/s. **b** Electrochemical impedance spectra of Co_3_O_4_NPs/df-GNS and Co_3_O_4_NWs/df-GNS electrodes obtained by applying a sine wave with amplitude of 5.0 mV over the frequency range 100 kHz~0.1 Hz

In addition to the galvanostatic charge-discharge tests, the cyclic voltammograms (CV) of the fabricated Co_3_O_4_NWs/df-GNS were presented in Fig. 7a. In the first cycle, two cathodic peaks were observed at voltage range ~ 1.1 and ~ 0.4 V, which could be correlated to the multistep electrochemical reduction of Co_3_O_4_ by Li to give metallic Co (lithiation) [22]. The main anodic peak at 2.2 V is ascribable to the oxidation reaction of metallic Co to reform Co_3_O_4_. This reversible electrochemical conversion reaction can be summarized as follows:

$$ {\mathrm{Co}}_3{\mathrm{O}}_4+8\mathrm{Li}{\displaystyle \begin{array}{c}\mathrm{discharge}\\ {}\leftrightharpoons \\ {}\mathrm{charge}\end{array}}3\mathrm{Co}+4{\mathrm{Li}}_2\mathrm{O} $$

An enormous strong cathodic peak at ~ 0.4 V in the first cycle was observed; however, cathodic peak in the second and third cycle at this voltage region only emerged as a “bump.” We conclude this to the formation of a solid-electrolyte-interface (SEI) film during the first discharge process, which remained stable in the subsequent cycles. As a result, the CV curves of the second and third cycles almost overlapped on each other, showing cathodic peaks at 1.2 and 0.7 V and broad anodic peak at 2.1 V. This behavior suggested the stabilized SEI layers and high electrochemical reversibility of Co_3_O_4_NWs/df-GNS electrodes. The Co_3_O_4_NWs/df-GNS and the Co_3_O_4_NPs/df-GNS electrodes were also characterized by electrochemical impedance spectroscopy (EIS). The Nyquist plots of two electrodes, shown in Fig. 7b, both exhibits a semicircle in the high-frequency region and a sloped line in the low-frequency region. The impedance data can be fitted in equivalent electrical circuit shown in the inset of Fig. 7b, in which CPE is the constant phase element related to double-layer capacitance, We is the Warburg impedance, and Rs and Rct represents the resistance of electrochemical system and the charge transfer resistance, respectively. The charge transfer resistance Rct of Co_3_O_4_NWs/df-GNS was calculated to be 52.6 Ω; however, that of Co_3_O_4_NPs/df-GNS was 109 Ω. We suggest this superior charge transfer capability of the nanowires contributed to the rate performance of the electrode.

## Conclusions

In summary, we proposed a novel, facile, and environmental-friendly strategy to synthesize Co_3_O_4_NWs with superior efficiency and cost-effectiveness. Excellent lithium storage properties were observed in such nanomaterials. The XRD and SAED results indicated that the as-obtained Co_3_O_4_NW samples displayed good quality in chemical and phase composition. The Co_3_O_4_NWs with the average diameter approximately 180 nm and the length ranging from 3 to 8 μm were observed by SEM and TEM. These nanowires exhibit good electrochemical performance, achieving lithium storage capacity higher than 700 mAh/g, as a result of the fast electron transport and volume-change-accommodating nature of the unique 1D-2D hybrid nanostructure in conjunction with 2D graphene.

## Abbreviations

Co_3_O_4_: Cobalt oxides
Co_3_O_4_NPs: Co_3_O_4_ nanoparticles
Co_3_O_4_NWs: Co_3_O_4_ nanowires
CV: Cyclic voltammograms
DEC: Diethyl carbonate
df-GNS: Defect-free graphene nanosheets
EC: Ethylene carbonate
EIS: Electrochemical impedance spectroscopy
GNS: Graphene nanosheets
HRTEM: High-resolution TEM
LIBs: Lithium-ion batteries
NMP: *N*-Methyl-pyrrolidone
PC: Propylene carbonate
SAED: Selected area electron diffraction
SEI: Solid electrolyte interface
SEM: Emission scanning electron microscope
TEM: Transmission electron microscopy
XRD: X-ray diffraction analysis
